# Efficacy of the mermithid nematode, *Romanomermis iyengari,* for the biocontrol of *Anopheles gambiae*, the major malaria vector in sub-Saharan Africa

**DOI:** 10.1186/s13071-019-3508-6

**Published:** 2019-05-22

**Authors:** Ayaba Z. Abagli, Thiery B. C. Alavo, Rafael Perez-Pacheco, Edward G. Platzer

**Affiliations:** 1 Laboratoire d’Entomology Appliquée/Centre Edward Platzer, Université d’Abomey-Calavi, BP:215, Godomey, Benin; 2 CIIDIR Oaxaca, Instituto Politécnico Nacional, Xoxocotlan, Oaxaca, 71230 Mexico; 3 Department of Nematology, University of California, Riverside, CA 92521-0415 USA

**Keywords:** *Anopheles gambiae*, Malaria, Entomopathogenic nematodes, *Romanomermis iyengari*, Vector control

## Abstract

**Background:**

The intensive use of chemical insecticides against mosquitoes has led to the development of widespread insecticide resistance. Control of *Anopheles* mosquitoes in malaria endemic areas of sub-Saharan Africa has become increasingly difficult. There is an urgent need for malaria control programmes to adopt more integrated mosquito management approaches that include sustainable, nonchemical solutions. The mermithid nematode *Romanomermis iyengari* is one of several natural control alternatives to synthetic pesticides for mosquito suppression. This study evaluated the effectiveness of the nematode *R. iyengari* for control of *Anopheles gambiae*.

**Methods:**

The nematode *R. iyengari* was mass-produced, and pre-parasitic stage (J2) were used for laboratory and field experiments. In laboratory experiments, two concentrations of pre-parasitics (5 and 10 J2 per larva) were tested against first- (L1), second- (L2) and third-instar (L3) larvae of *An. gambiae*. Infected larvae were observed daily to determine their mortality rate and the number of post-parasitic nematodes emerging from dead larvae. In field experiments, 3500, 4000 and 5000 J2/m^2^ were sprayed in separate natural *Anopheles* breeding sites. After treatment, the larval mosquito density in the breeding sites was assessed every 5–7 days.

**Results:**

Laboratory results showed that larval *An. gambiae* is susceptible to nematode infection: 100% L1 larvae died within 24 hours post-treatment, and 100% of both L2 and L3 larvae died within 7 days, regardless of nematode concentrations. The average number of post-parasitic nematodes emerging per larva increased with increasing nematode concentration. In field experiments, the monthly applications of 3500 to 5000 pre-parasitic nematodes per m^2^ eliminated larval mosquito development in *Anopheles*- and mixed breeding sites. Larval mosquito density dramatically decreased five days after the first treatment in all treated sites and was maintained at a very low level during the whole experimental period. Basically, only early instar larva were detected in treated sites throughout the test period. The average number of post-parasitic nematodes emerging per larva collected in treated sites was 1.45, 2, and 5.7 respectively for sites treated with 3500, 4000, and 5000 J2/m^2^.

**Conclusions:**

Malaria mosquito larvae is susceptible to *R. iyengari* infection in West Africa. Parasitism intensity depends on tested nematode concentrations. Monthly application of 3500 J2/ m^2^ was enough to control effectively larval *An. gambiae* in wetlands and floodable locations in West Africa.

## Background

Mosquitoes are disease-carrying insects for chikungunya, dengue, yellow fever, and malaria which remains the deadliest disease worldwide [1, 2]. In the fight against malaria vectors, synthetic insecticides have been extensively used either for spraying breeding sites and homes, or for impregnating mosquito nets and curtains. The intensive use of chemical insecticides has led to the emergence of a resistance to control agents [3]. This resistance occurred for the organochlorine DDT, well-known for its shock effect and extreme persistence [4]. This has made the fight against mosquitoes increasingly difficult. While the immediate toxicity of most of the chemicals used is generally low, their continuous use over long periods can cause serious harm to public health, especially with regard to fertility and cancer [5, 6].

Given these problems, it is urgent to consider the use of alternative means for vector control. In this way, natural enemies of mosquitoes can be considered. *Romanomermis iyengari* (Mermithidae) is one of several species of entomopathogenic nematodes which parasitize and kill mosquito larvae [7].

Initial information on *R. iyengari* probably came from observations made by Ross [8] who reported the presence of mermithid nematodes in a sample of *Culex* larvae in India. Iyengar [9] found juvenile mermithid nematode parasites in the larvae of seven different species of anopheles. These nematodes were later described by Welch [10] as *R. iyengari.*

Various studies have demonstrated the effectiveness of these nematodes for mosquito control in some parts of the world. In Tajikistan, Vladimirova et al. [11] demonstrated that *R. culicivorax* and *R. iyengari* were much more effective against *Anopheles* than *Culex* mosquitoes. In Uzbekistan, Pridantseva et al. [12] demonstrated *Anopheles* infection with *R. iyengari*. Studies on mosquitoes conducted in Azerbaijan showed that *R. iyengari* infects both *An. sacharovi* and *Cx. theileri* [13]. The results of field work carried out in Cuba were particularly encouraging. Santamarina et al. [14] demonstrated that for a concentration of 1000 pre-parasitic *R. iyengari* per square metre of surface area, the percentage of larval reduction was 80–100% for *Anopheles*. In Mexico, a dose of 2000–3000 pre-parasitic juvenile *R. iyengari* per square metre produced an infection rate of approximately 85–100% in *An. pseudopunctipennis* larvae [15]. Similar results were also obtained in Brazil, where 12 natural sites of *Anopheles* were treated with 2000 infectious nematodes per square metre of surface. After a week, *An. albitarsis* and *An. rondoni* populations were reduced by 85–97% [16]. Despite the effectiveness of *R. iyengari* in regions where the climate is comparable to that of malaria endemic countries, this nematode has never been used in experiments in Africa, which accounts for 91% of deaths caused by malaria [17].

The present study has evaluated the potential of the entomopathogenic nematode *R. iyengari* for the biological control of *Anopheles gambiae*, the major vector of malaria in Africa.

## Methods

### Nematodes production

Production of *R. iyengari* in Benin [18] was initiated with eggs obtained from the Department of Nematology (University of California, Riverside, USA). *Culex quinquefasciatus* was used as host for the nematodes. Chickens (*Gallus domesticus*) were used to supply these mosquitoes with blood meals. A small plastic tray (12 × 8 × 6 cm) containing about 500 ml of water was provided in each mosquito cage for oviposition.

Four days after a blood meal, the egg rafts which contained about 120 eggs each, were collected and 6 of these were deposited in a plastic tray (25 × 15 × 12 cm) containing 2 l water. Five hundred plastic trays were installed on metal shelves in the nematode production room where temperature and relative humidity (RH) were 28 ± 2 °C and 70–90%, respectively. The containers were covered with a mesh screen to prevent oviposition by wild mosquitoes. When the larvae reached the second-instar stage (2 days), they were infected with preparasitic nematodes (second-stage juveniles, J2) of *R. iyengari*. The infection ratio was 3 J2 per mosquito larva. The nematodes were taken from cultures that had been stored for 8 weeks. These cultures were flooded with sterilised (chlorine-free) water to induce the eclosion of eggs and emergence of infective preparasites from the substrate. Fourteen hours after flooding the cultures (overnight), the water was decanted, and the concentration of nematodes in the water was calculated by volumetric dilution. After 8 days, the mosquito larva died and floated on the surface, indicating the end of the parasitic phase of the nematode. The water containing post-parasitic juveniles (J4) together with the dead mosquito larva and other debris was poured onto a sieve. The sieve containing the larvae and J4 was then placed in a container with clean water. After a few minutes, the J4 passed through the sieve into the clean water so that when the sieve was removed, the dead mosquito larva and debris were separated from the J4 that settled on the bottom of the container. The J4 were collected using a syringe and transferred to a glass beaker with clean water. The J4 were then washed thoroughly several times by sedimentation. Two grams (wet weight) of washed J4 (composed of 457.6 ± 1.38 females and 583 ± 0.59 males, as determined in 25 samples) were deposited in round plastic containers (13 cm diameter) with previously sterilised coconut coir fibres (35 g) and 500 ml sterilized (chlorine-free) water. About 3 h later (when all nematodes had moved into the substrate), the water was decanted, and the containers covered and stored for eight weeks so that the nematodes could reach sexual maturity, mate and deposit eggs. They were kept in a climate-controlled room at 27 ± 2 °C and 85% RH. Every week, condensation water droplets were removed from the containers with cotton tissue to prevent premature hatching of nematodes eggs.

### Laboratory test for mosquito larvae susceptibility to *R. iyengari* nematode

To determine whether *R. iyengari* can infect and kill *An. gambiae*, tests were performed on the first three larval stages (L1, L2 and L3). The larvae used for these tests were collected in natural breeding sites of *An. gambiae* in Cotonou, Benin. Wild larvae were used to ensure the results could be extrapolated to field conditions.

The larvae were harvested on the day the tests were conducted. Once in the laboratory, the larvae were sorted and grouped by larval stage. For each stage, two batches of 100 larvae were established; one batch was infected with pre-parasitic nematodes and the other served as the untreated control group (zero nematode). Fish food was used to feed the experimental larvae throughout the trials. The larvae were incubated in 500 ml of distilled water.

The volumetric dilution method [19] was used to determine the concentration of suspended pre-parasitic nematodes. Concentrations of 5 and 10 pre-parasitic juvenile nematodes per larva were used for the tests.

Four days after infection, 20 larvae were transferred individually into Petri dishes containing 30 ml of distilled water for daily observation. The experimental larvae were kept in a room where the temperature is maintained at 27 °C and observed daily under a stereomicroscope to determine the mortality rate and number of post-parasitic nematodes emerging from dead larvae.

### Efficacy tests of *R. iyengari* against mosquito larvae in a natural setting

#### Description of treated sites

Surveys were conducted in various districts to identify habitats of *Anopheles* larvae to be used for the experiments. Three different breeding sites were selected for the tests.

*First site*: yard of a house located in the 13th district of Cotonou, Benin (West Africa). In this yard, rainwater had stagnated for several months; the water was clear and covered an area of 150–320 m^2^ (depending on the rainfall). The water at this site was 46.66 cm deep on average and contained only *An. gambiae* larvae.

*Second site*: yard of another house located in the 13th district of Cotonou, where rainwater had also stagnated for several months. The water at this site covered an area of 20–50 m^2^ (depending on the rainfall) and was 32.33 cm deep on average; the water was clean but contained organic waste in some places. It was a mixed site containing *An. gambiae* and *Cx. quinquefasciatus* larvae.

*Third site*: flooded and uninhabited two-room hut, located in the 6th district of Cotonou. The water at the site was clear and covered an area of 20 m^2^ and was 30 cm deep on average. This site contained only *An. gambiae* larvae.

The physical and chemical parameters of these sites, i.e. temperature, pH, conductivity, salinity, redox percentage (POR) and total dissolved solids (TDS) are presented in Table 1.

**Table 1:** Physical and chemical parameters of *Anopheles* and mixed breeding sites treated with *R. iyengari*

	Site 1	Site 2	Site 3
Temperature (°C)	31.3	31.6	26.8
pH	9.49	9.85	7.99
Salinity (mg/l)	0	0	0.3
Conductivity (µS/cm)	333	317	996
POR (mV)	− 135	− 152	− 57
TDS (mg/l)	164	156	496

#### Experimental procedure

Doses of 3500, 4000 and 5000 pre-parasitic nematodes (juvenile 2, J2) per square metre were applied at sites 1, 2 and 3, respectively. The nematodes were applied once a month at the three sites throughout the rainy season (approximately 5 months).

At each field site, the required nematode suspension was applied over the entire site with a knapsack sprayer. Immediately prior to each application, the larval mosquito density was measured at each site. A total of 3 liters of water was collected at different areas of the site with a 100 ml dipper. Using a Pasteur pipette, the number of larvae was counted and averaged to obtain the larval density expressed as the number of larvae per litre of water. After nematode spraying, the larval density in each site was measured at 5- or 7-day intervals to determine the larval mosquito population dynamics during the experimental period.

Additionally, five days after the first nematode application, at least 20 mosquito larvae were collected at each treated site and subsequently examined in the laboratory to determine if sprayed nematode had effectively infected the larvae. The sampled larvae were transferred individually into Petri dishes and observed daily under a stereomicroscope to determine their mortality rate and the infection intensity (number of post-parasitic nematodes emerging per larva).

### Statistical analyses

For the laboratory tests, probit analysis was performed to determine if there was a significant difference between tested nematodes concentrations. Pearsonʼs correlation test was also performed to determine the correlation between nematode concentration and number of post-parasitic nematodes emerging per larva. The SPSS statistics package was used to perform the probit analysis and the correlation test.

For field experiments, the average density of mosquito larvae was calculated for each day of observation and the population dynamics of larval mosquitoes in treated sites was determined. The percentage reduction of mosquito larvae compared to the situation before the first treatment was also calculated for each day of observation; for this, the following formula was used [20]:

$$ {\text{Percentage reduction}} = \left[ {\left( {{\text{LDpre}} - {\text{LDpost}}} \right)/{\text{LDpre}}} \right] \times 100 $$

where LDpre is the larval density before treatment and LDpost is the larval density after treatment.

In addition, a mixed analysis of variance (ANOVA) on repeated measures was performed on the numbers of larvae counted after nematode spraying. This analysis was preferred over the traditional ANOVA because of a possible dependence between the number of larvae counted during an observation and that of a following one, thus violating one of the ANOVA conditions (independence between observations). In this model, the sites (which received different doses of pre-parasitic nematodes) were taken as the random factor, while the stages of larval development (L1, L2, L3, L4 and pupae) were considered the fixed factor. SASv9.2 software was used to perform the ANOVA test on repeated measures.

## Results

### Susceptibility of *An. gambiae* to nematode infection

The laboratory test showed that all L1 larvae died within 24 h after infection at an infection ratio of 10 J2 nematodes per larva, i.e. a mortality rate of 100%. For L2 larvae, the mortality rates were 0%, 30%, 75% and 100%, respectively, at the 4th, 5th, 6th and 7th day after treatment. Regarding L3 larvae, the rates were 0%, 25%, 90% and 100%, respectively, at the 4th, 5th, 6th and 7th day after treatment.

At an infection ratio of 5 J2 per larva, 100% of the L1 larvae were also killed within 24 h post-infection. For L2 larvae, the mortality rates were 0%, 15%, 90% and 100%, respectively, at the 4th, 5th, 6th and 7th day after treatment. Mortality rates for the L3 larvae were 0%, 15%, 65% and 100%, respectively, at the 4th, 5th, 6th and 7th day after treatment. No mortality was recorded in the control larvae during the laboratory tests.

Statistical analysis revealed no significant difference between the two tested nematode concentrations. For L2 larvae, the lethal times (LT_50_) were 5.45 and 5.46 days, respectively, for 5 and 10 nematodes per larva (*χ*^2^ = 19.56, *df* = 15, *P* = 0.189). Regarding L3 larvae, the LT_50_ were 5.70 and 5.35 days, respectively, for 5 and 10 nematodes per larva (*χ*^2^ = 10.45, *df* = 15; *P* = 0.791).

**Fig. 1:**
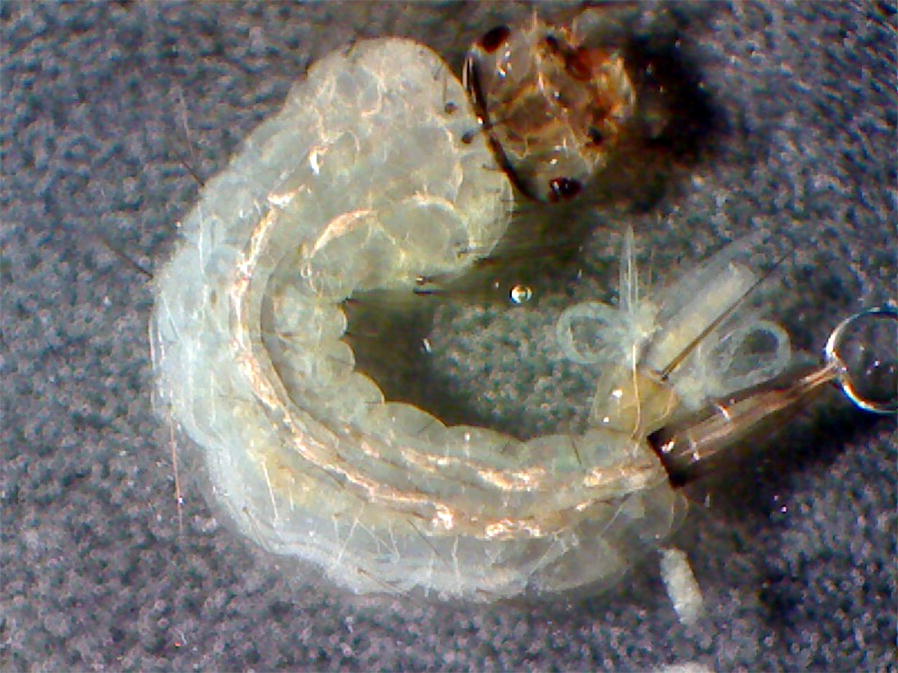
Dead larvae with post-parasitic nematode emergence

Table 2 shows the average number of post-parasitic nematodes emerging per larva for each larval stage of *An. gambiae.* No post-parasitic nematode emerged from L1 larvae regardless of the concentrations tested, and the L2 larvae generated more post-parasitic nematodes than the L3 larvae. Since the mortality rate of L1 larvae was 100% within 24 h after treatment, nematodes did not have sufficient time to complete their development and died therefore with the larvae. For L2 and L3 stages, nematodes developed normally within the larvae. These larvae died when post-parasitic nematodes started emerging (Fig. 1).

**Table 2:** Average number of post-parasitic nematodes emerging per larval *An. gambiae* treated in laboratory

Infectious nematodes concentration (J2 per larva)	Mean number ± SD of post-parasitic nematodes emerging per larva at each larval stage		
	L1	L2	L3
10	0	7.6 ± 3.2	6.8 ± 4.4
5	0	4.7 ± 3.3	4.15 ± 2.3

*Abbreviation*: SD, standard deviation

### Effectiveness of the *R. iyengari* against *An. gambiae* in the field

#### Effects of the dose of 3500 nematodes per m^2^

**Fig. 2:**
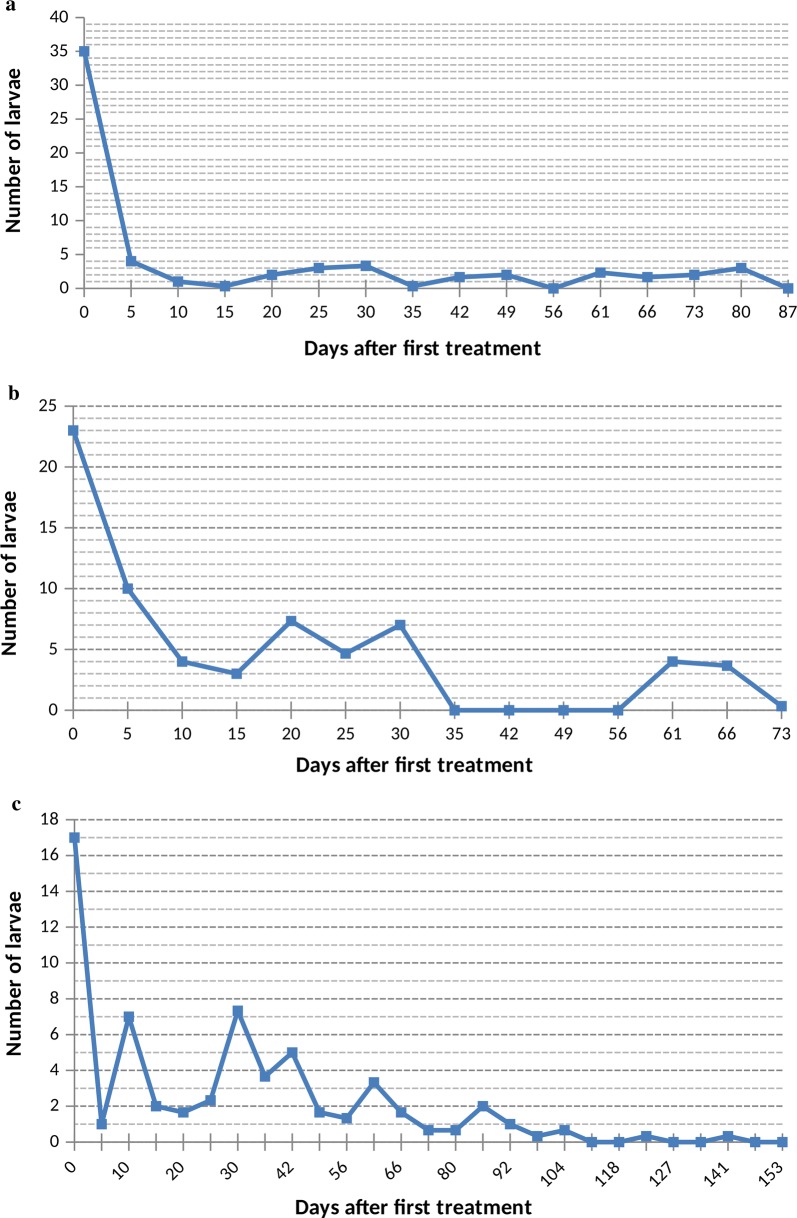
Larval mosquito population dynamics in breeding sites treated with nematodes. **a** 3500 juveniles 2 per m^2^. **b** 4000 juveniles 2. **c** 5000 juveniles 2

At the *Anopheles* site treated with 3500 pre-parasitic nematodes per square metre, the density of mosquito larvae dropped from 35 to 4 larvae per liter of water five days after the first treatment. Over the entire experiment period, larval density of *Anopheles* varied between 0 and 4 larvae per liter of water (Fig. 2a). Throughout the test period, the majority of larvae collected at this site were L1 and L2 larvae. Later stage larvae and pupae became very rare 5 days after the first treatment.

Daily observations in the laboratory of larvae sampled at this site 5 days after the first treatment revealed larval mortality rates of 0%, 0%, 20%, 40%, 50%, respectively, at the 5th, 6th, 7th, 8th and 9th day after treatment. The average number of post-parasitic nematodes emerging per larva was 1.45. These results confirm that the reduction in larval density recorded at this site can be attributed to the effect of *R. iyengari*.

#### Effects of the dose of 4000 nematodes per m^2^

The mixed site (containing both *Anopheles* and *Culex* larvae) was treated with 4000 J2 nematodes per square metre. Five days after the first treatment, the density of mosquito larvae dropped from 23 to 10 larvae per liter of water. Over the entire experiment period, larval density at this site varied between 0–7.33 larvae per liter of water (Fig. 2b). Throughout the test period, the majority of larvae sampled at this site were of L1 and L2 stages. Larvae of later stages and pupae also became very rare 5 days after the first treatment.

Daily observations in the laboratory of larvae sampled at this mixed site 5 days after the first treatment revealed larval mortality rates of 0%, 10%, 30%, 50% and 60%, respectively, at the 5th, 6th, 7th, 8th and 9th day after treatment. On average, 2 post-parasitic nematodes emerged per larva. Again, this confirms that the reduction in larval density recorded at this site can be attributed to the activity of *R. iyengari*.

#### Effects of the dose of 5000 nematodes per m^2^

At the *Anopheles* site treated with 5000 J2 per square metre, the density of mosquito larvae dropped from 17 to 1 larvae per litre of water five days after the first treatment. Throughout the test period, larval density at this site varied between 0–7.33 larvae per liter of water (Fig. 2c). Throughout the test duration, the majority of larvae sampled at this site were also L1- and L2-stage larvae. Here too, larvae at later stages and pupae became very rare 5 days after the first treatment.

Daily observations in the laboratory of larvae sampled at this site 5 days after the first treatment revealed larval mortality rates of 0%, 10%, 85% and 100%, respectively, at the 5th, 6th, 7th and 8th day after treatment, while the average number of post-parasitic nematodes emerging per larva was 5.7. These results also confirm that the reduction in larval density recorded at this site is also due to the action of *R. iyengari*.

Statistical analysis of larval densities observed at all treated sites showed that there is a highly significant difference between the number of larvae of different stages (L1, L2, L3 and L4), regardless of the length of time considered after first treatment of the sites (Table 3). This confirms that L1 and L2 larvae were dominant in the treated sites, indicating that these larvae which come from newly oviposited mosquito eggs did not have time to grow before being killed by *R. iyengari*.

**Table 3:** Results of analysis of variance used in testing for site and stage effects

Source	*df*	Type III SS	Mean square	*F*-value	*P*-value
Site	2	5.5740741	2.7870370	0.71	0.5298
Stage	2	512.2345679	170.7448560	43.34	0.0002
Error	6	23.6358025	3.9393004		

## Discussion

This study has assessed the susceptibility of *An. gambiae* larvae to *R. iyengari*. Our results show that this nematode parasitizes and effectively kills larvae of *An. gambiae*, and the parasitic intensity (the number of nematodes parasitizing a larva) depends on the concentration of nematodes. The mortality rates observed for each larval stage indicate that L1 and L2 larvae are more susceptible to infection by the nematode, compared to later stage larvae. Authors who obtained similar results have attributed this to the fact that the thin cuticle of young larvae facilitates the invasion of pre-parasitic nematodes, which have more difficulty penetrating the thicker cuticles of older larvae [21–24].

Regardless of the nematode concentrations tested (10 or 5 J2 per larva), all L1 *An. gambiae* larvae died 24 hours after the infection, and 7 days were sufficient for later stage larvae to be killed by *R. iyengari*. These results indicate that *R. iyengari* can be effective in controlling this mosquito species in natural settings in Benin, West Africa.

In the field, larval density in the *Anopheles* and mixed breeding sites fell sharply 5 days after the nematode application. The larvae collected at treated sites and incubated in the laboratory also showed significant mortality with post-parasitic nematodes emerging from dead larvae. These data clearly indicate that the drastic drop in larval density observed in these sites 5 days after treatment is due to the parasitic nematode.

Various authors have reported similar levels of larval density reduction following the release of *R. iyengari* in countries such as Cuba and Mexico [14–16, 20, 21, 25–28]. Distinctive feature of the present study is that various nematode concentrations were tested, and results showed that there was no significant difference between these concentrations in the *Anopheles* and the mixed breeding sites. Doses of 3500, 4000 and 5000 pre-parasitic nematodes had the same effect, namely, the elimination of larval populations in the treated sites. This also confirms the results obtained in laboratory where concentrations of 5 and 10 pre-parasitic nematodes per larva produced the same mortality levels. These results demonstrate that a low dose of *R. iyengari* per square metre should be sufficient to control *An. gambiae* in West Africa.

A monthly release of pre-parasitic nematodes in the *Anopheles* and mixed breeding sites has led to the elimination of larval populations of *An. gambiae,* a major malaria vector in West Africa. The physicochemical data of water in treated sites were in the range considered to be optimal for allowing *R. iyengari* to reproduce and to continue parasitizing larval mosquitoes over a long period [12, 26, 29–31]. Perez-Pacheco et al. [20] indicated that a number of topographical and hydrological factors can play role in the effectiveness of *R. iyengari* against malaria vectors. In experiments conducted in Mexico, these authors obtained higher larval population reduction in the breeding site with stagnant water and lower salinity, compared to breeding sites with flowing water and high salinity. In the present study, high larval population reduction was achieved in all treated sites where water was also stagnant and salinity was about 0 mg/l. We can therefore conclude that characteristics of mosquito breeding sites in South Benin are favourable for the use of *R. iyengari* in malaria vector control.

*Romanomermis iyengari* is a parasite specific to mosquito larvae and its mass production is well established [32–37]. A large-scale production system of this worm has been developed for Africa. It uses endogenous and cost-effective materials which are locally available in sub-Saharan Africa. The production system employs three technicians and can produce monthly a sufficient amount of nematodes to treat at least 75 000 square metres of breeding sites [18]. Therefore, the insect parasitic nematode *R. iyengari* could be easily used as a component of integrated mosquito control programmes in malaria endemic countries.

Furthermore, *R. iyengari* is harmless for vertebrates. In experiments conducted by Gajanana et al. [38], live infective juveniles of *R. iyengari* were injected intravenously into animals such as mice, guinea pig, rabbit and chicken; results showed that the overall health of these vertebrates remained unchanged. In treated animals, the histology of organs such as liver, spleen, kidneys, lungs and the gastrointestinal system revealed no lesions linked to the nematode infection. In addition, *Gambusia affinis* fish exposed over several days to a large number of infective juvenile worms were not affected in any way. Thus, the use of this biological control agent raises no concern for the health of vertebrates.

In Senegal, West Africa, a nematode of the family Mermithidae was found in adults of *An. gambiae*, *An. funestus* and *An. rufipes* [39]. Although the species involved has not been identified, this discovery confirms that West African ecosystems are favourable to mermithid nematodes. Consequently, we believe that enhanced biological control of malaria vectors using *R. iyengari* would be a great success in West Africa.

## Conclusions

Malaria mosquito larvae are susceptible to *R. iyengari* infection in West Africa. Parasitism intensity depends on tested nematode concentrations. Monthly application of 3500 J2/m^2^ was sufficient to control effectively larval *An. gambiae* in wetlands and floodable locations in West Africa.

## Data Availability

The datasets used and/or analysed during the present study will be made available by the corresponding author upon reasonable request.
